# Immunopsychiatry of late life depression: role of ageing-related immune/inflammatory processes in the development and progression of depression

**DOI:** 10.1017/neu.2025.10019

**Published:** 2025-06-13

**Authors:** Antonio L. Teixeira, Izabela G. Barbosa, Moises E. Bauer, Aline S. de Miranda

**Affiliations:** 1 Geriatric Neuropsychiatry Division, The Glenn Biggs Institute for Alzheimer’s & Neurodegenerative Disease, The University of Texas Health Science Center at San Antonio, San Antonio, TX, USA; 2 Department of Psychiatry, Medical School, Universidade Federal de Minas Gerais, Belo Horizonte, Brazil; 3 Laboratory of Immunobiology, School of Health and Life Sciences, Pontifical Catholic University of Rio Grande do Sul (PUCRS), Porto Alegre, Brazil; 4 Laboratory of Neurobiology, Department of Morphology, Institute of Biological Science, Federal University of Minas Gerais, Belo Horizonte, MG, Brazil

**Keywords:** Depression, late life depression, vascular depression, neurodegeneration, neuroinflammation

## Abstract

**Background:**

Late-life depression (LLD) arises from a complex interplay among biological, psychological, and social factors. Biologically, three main hypotheses have been proposed to explain the distinct clinical features of LLD. The vascular hypothesis supports vascular-related white matter changes in the development of LLD, while the neurodegenerative hypothesis suggests that LLD might be a prodrome of neurodegenerative diseases. The inflammatory hypothesis, which is the main focus of this review, posits that heightened inflammation underlies LLD directly or indirectly through neurodegenerative and microvascular alterations.

**Methods:**

This is a non-systematic review on the role played by inflammation in the pathophysiology of LLD and the related opportunities to define biomarkers and therapeutic targets. We searched PubMed from January 2010 through March 2025 for relevant English-language studies.

**Results:**

Patients with LLD have elevated circulating levels of inflammatory biomarkers (e.g., C-reactive protein and interleukin-6) as well as evidence of neuroinflammation. Although the exact origin of this inflammatory profile remains unclear, it is thought to be exacerbated by immune cell senescence and the presence of physical comorbidities, including cardiovascular and metabolic diseases. Pharmacological (e.g., selective serotonin receptor inhibitors) and non-pharmacological (e.g., diet, physical interventions) approaches for LLD seem to exert their therapeutic effect, at least in part, through inflammation-related mechanisms.

**Conclusion:**

Recognizing the unique features of LLD compared to depression in other periods of life is an important step toward its proper management. More specifically, understanding the role of inflammation in LLD holds both theoretical and practical implications, including anti-inflammatory or immune-based strategies as potential therapeutic interventions.

## Graphical abstract

**Figure:**
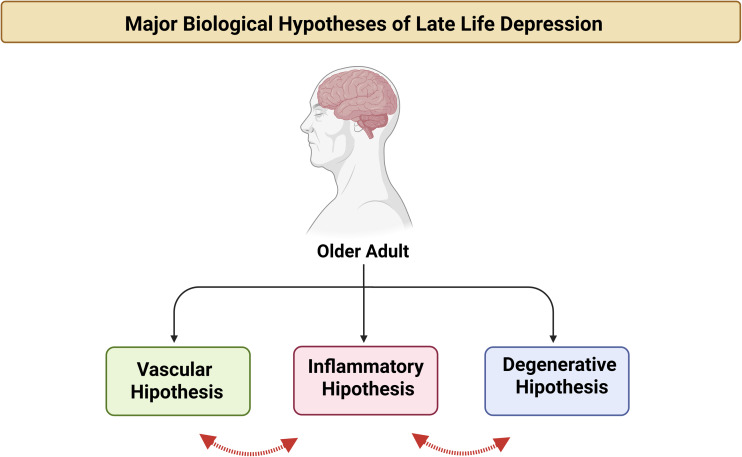

Highlights

- LLD has unique clinical and biological features compared to depression in other periods of life.
- Low-grade systemic inflammation or inflammaging may underlie LLD onset and progression.
- Targeting aging-associated immune/inflammatory changes might be a promising therapeutic approach for LLD.

Summations

- Late life depression is associated with low-grade systemic inflammation or inflammaging.
- The pro-inflammatory profile may differ between early-onset versus late-onset depression.
- Diet and microbiota-targeted interventions may be promising in attenuating depression-related inflammation.

Considerations

- While the pathogenesis of the low-grade inflammation in LLD is still unclear, it is potentiated by the senescence of immune cells (that tend to assume a proinflammatory profile with ageing) and the burden of physical comorbidities like cardiovascular and metabolic diseases.
- To date studies have failed to report any meaningful differences in the pro-inflammatory profile between early-onset and late life depression.
- The majority of studies conducted in the field of nutrition psychiatry was with adult population. The benefits of diet and microbiota-target interventions in older adults and LLD remain to be established.

## Introduction

Depressive disorders are one of the most common mental disorders across the lifespan. Late-life depression (LLD) refers to depressive disorders occurring in individuals aged 60 years or older. It encompasses a spectrum of depressive conditions, including major depressive disorder (MDD), persistent depressive disorder (dysthymia), and subsyndromal depression (American Psychiatric Association, 2013). LLD can either be a late-onset depression (LOD), when the first lifetime depressive episode began after age 60, or an early-onset depression (EOD), meaning that an older adult has experienced recurrent depressive episodes with a first episode occurring earlier in life. Depression is often perceived as an expected psychological reaction to the challenges associated with ageing. Older adults are commonly portrayed as depressed, lonely, and preoccupied with impending disability and death. However, older age is associated with increased subjective wellbeing and decreased negative affect. Older adults are also more likely to anticipate the occurrence of adverse events and rehearse the experience in their mind, which may lessen the psychological and emotional impact of the event, supporting the view that depression must not be seen as an unequivocal outcome of ageing (Richardson *et al*., 2023).

The cross-sectional prevalence of MDD is estimated to be 3.8% in the whole population, affecting 5% of adults, and around 6% of adults older than 60 years based on representative surveys, medical data and statistical modelling (“Homepage | Institute for Health Metrics and Evaluation”, n.d.; Kok & Reynolds, 2017). The rates of depressive disorders increase significantly in the oldest age groups, reaching numbers above 20% from 85 to 89 years-old and above 30% in 90 years and older (Corneliusson *et al*., 2024). Epidemiological studies have identified several predictors of LLD, including the paucity of leisure and intellectual activities, scarcity of social activities, poor physical health, unhealthy lifestyle, and negative views on ageing (Read *et al*., 2017; Maier *et al*., 2021; Belvederi Murri *et al*., 2022). While a complex interplay among biological, psychological, and social factors plays a role in LLD, the increase of prevalence rates with rising age may be explained by ageing-related factors such as a higher proportion of females, more physical disability, higher cognitive impairment, lower socioeconomic status, and stay in hospital and nursing homes (Luppa *et al*., 2012; Cheung & Mui, 2023).

The negative association between LLD and quality of life has been confirmed by cross-sectional and longitudinal epidemiological studies (Sivertsen *et al*., 2015). Depressive disorders in the elderly also present a reciprocal interaction with frailty, a geriatric syndrome conceptualised as a clinical state of poor health in which there is an increased vulnerability to functional dependency and/or mortality when exposed to stressors (Soysal *et al*., 2017; Zou *et al*., 2023; Kim & Rockwood, 2024). Accordingly, LLD is associated with 1.34 increased risk of all-cause mortality, and 1.31 cardiovascular mortality in elderly people (Wei *et al*., 2019). There is also strong association between depression and dementia. However, the nature of this association, i.e. whether depression is a risk factor, a prodrome or a consequence of a neurodegenerative disease progressing with dementia, remains debateable (Byers & Yaffe, 2011; Antonio L. Teixeira *et al*., 2025a).

LLD is a complex and multifaceted condition. The cellular and molecular mechanisms underlying LDD pathophysiology remain to be clearly determined (Xia *et al*., 2023). The traditional monoamine neurotransmitter hypothesis fails to explain the onset and progression of LLD, as partly reflected on the limited benefits of antidepressants for its treatment (Tedeschini *et al*., 2011; Alexopoulos, 2019; Patrick *et al*., 2024a; de Miranda *et al*., 2025). Therefore, exploring new pathophysiological hypotheses and therapeutic approaches for LLD is highly needed (Xia *et al*., 2023). Growing evidence has supported the link between ageing-related inflammation and LLD (Diniz *et al*., 2017; Charlton *et al*., 2018; Kim *et al*., 2018; Dias *et al*., 2024). Unravelling the relationship between inflammation and LLD could lead to novel diagnostic and prognostic biomarkers and new treatments based on immune and/or anti-inflammatory strategies for this disabling condition.

The objective of this manuscript is to review the physiopathology of LLD highlighting the role played by increased inflammation and the related opportunities to define biomarkers and therapeutic targets.

## The physiopathology of late-life depression

The three major biological hypotheses proposed to explain depression in older adults are 1) the “inflammatory hypothesis”, which suggests that LLD can be driven by ageing-related increase in inflammation; 2) the “vascular hypothesis”, which implicates vascular-related white matter lesions in the development of LLD; and 3) the “degenerative hypothesis”, which suggests that an emerging depression in an older adult can be the prodrome of a neurodegenerative disease. These hypotheses are not mutually exclusive and highlight the pivotal role of inflammation in LLD. While inflammation constitutes the basis of the “inflammatory hypothesis” of LLD, inflammatory mechanisms and pathways have been implicated in the development of neurodegenerative and microvascular (white matter) changes, as discussed below.

### Immune dysfunction in late-life depression

There are several lines of evidence supporting the association between mood disorders, especially MDD, and immune dysfunction toward a pro-inflammatory profile (Colpo *et al*., 2018; Bauer & Teixeira, 2019; Brunoni *et al*., 2020; Osimo *et al*., 2020; Islam *et al*., 2023). (Bauer and Teixeira, 2019) The most robust evidence comes from studies describing increased circulating levels of inflammatory markers in depression across the lifespan (Colpo *et al*., 2018; Brunoni *et al*., 2020). High levels of C-reactive protein (CRP), interleukin-6 (IL-6), and tumour necrosis factor (TNF-α), among other inflammatory mediators, have been reported in patients with depression (Haapakoski *et al*., 2015; Osimo *et al*., 2020; Islam *et al*., 2023). The most recent meta-analysis including a total of 2,708 participants, being 1,366 MDD drug-naïve patients and 1,342 controls, reported increased peripheral levels of TNF-α but not IL-6 and CRP (Islam *et al*., 2023), pointing out to the heterogeneity of results, possibly related to the intrinsic heterogeneity of MDD and confounding factors (e.g., physical comorbidities, lifestyle parameters). While not present in all patients with depression, at least a subgroup will exhibit this low-grade systemic inflammation (Teixeira *et al*., 2022). Interestingly enough, pharmacological treatment with antidepressants can decrease the circulating levels of inflammatory markers like IL-6, TNF-α, and CCL2 in depressed patients (Köhler *et al*., 2018; Wang *et al*., 2019). In this scenario, it is tempting to speculate whether there is an ‘inflammation-related subtype’ of depression with a specific pattern of therapeutic response. However, discrimination between atypical versus melancholic and moderate versus severe forms of depression does not seem possible based on inflammatory biomarkers (Veltman *et al*., 2018). In parallel with peripheral changes, neuroinflammation or an enhanced inflammatory profile in the central nervous system (CNS) is observed in patients with depression. For instance, MDD has been associated with high levels of IL-6, IL-8 and TNF-α in the cerebrospinal fluid (CSF) and increased TNF-α, monocytes chemoattractant protein 1 (MCP-1/CCL2) and toll like receptor 3 and 4 expression in *post-mortem* brain specimens (Torres-Platas *et al*., 2014; Enache *et al*., 2019; Pandey *et al*., 2019; Wang *et al*., 2019).

LLD has been associated with higher peripheral levels of inflammatory mediators/molecules, such as IL-6 (Dentino *et al*., 1999; Bremmer *et al*., 2008; Charlton *et al*., 2018; Nie *et al*., 2021; Lozupone *et al*., 2023; Dias *et al*., 2024), sTNFR2 (Diniz *et al*., 2010), GDF-15 (Teunissen *et al*., 2016), IL-1β (Thomas *et al*., 2005; Charlton *et al*., 2018; Kim *et al*., 2018) compared to non-depressed older adults. One important issue when studying LLD is that ageing itself has been associated with increased systemic inflammation, a phenomenon termed ‘inflammaging’. Franceschi and coworkers (2018) proposed that the physiological and unavoidable inflammatory tone increases over time and can become highly detrimental with ageing. Accordingly, all major age-related diseases would be derived from a basic ageing mechanism dependent on inflammation as the major player (Franceschi *et al*., 2018). Another relevant question is whether the pro-inflammatory profiles differ between EOD versus LOD. Most studies focused on LDD without discriminating EOD versus LOD (Diniz *et al*., 2016; Teunissen *et al*., 2016; Saraykar *et al*., 2018) or were not capable to observe meaningful differences between EOD and LOD (Kim *et al*., 2018; Rozing *et al*., 2019; Lozupone *et al*., 2023). Interestingly, patients with LLD exhibit a negative correlation between serum levels of TNF-α and hippocampal volume and cognitive performance (processing speed), suggesting that peripheral inflammation may also contribute to LLD-related CNS dysfunction (Ho *et al*., 2024).

**Figure 1.**
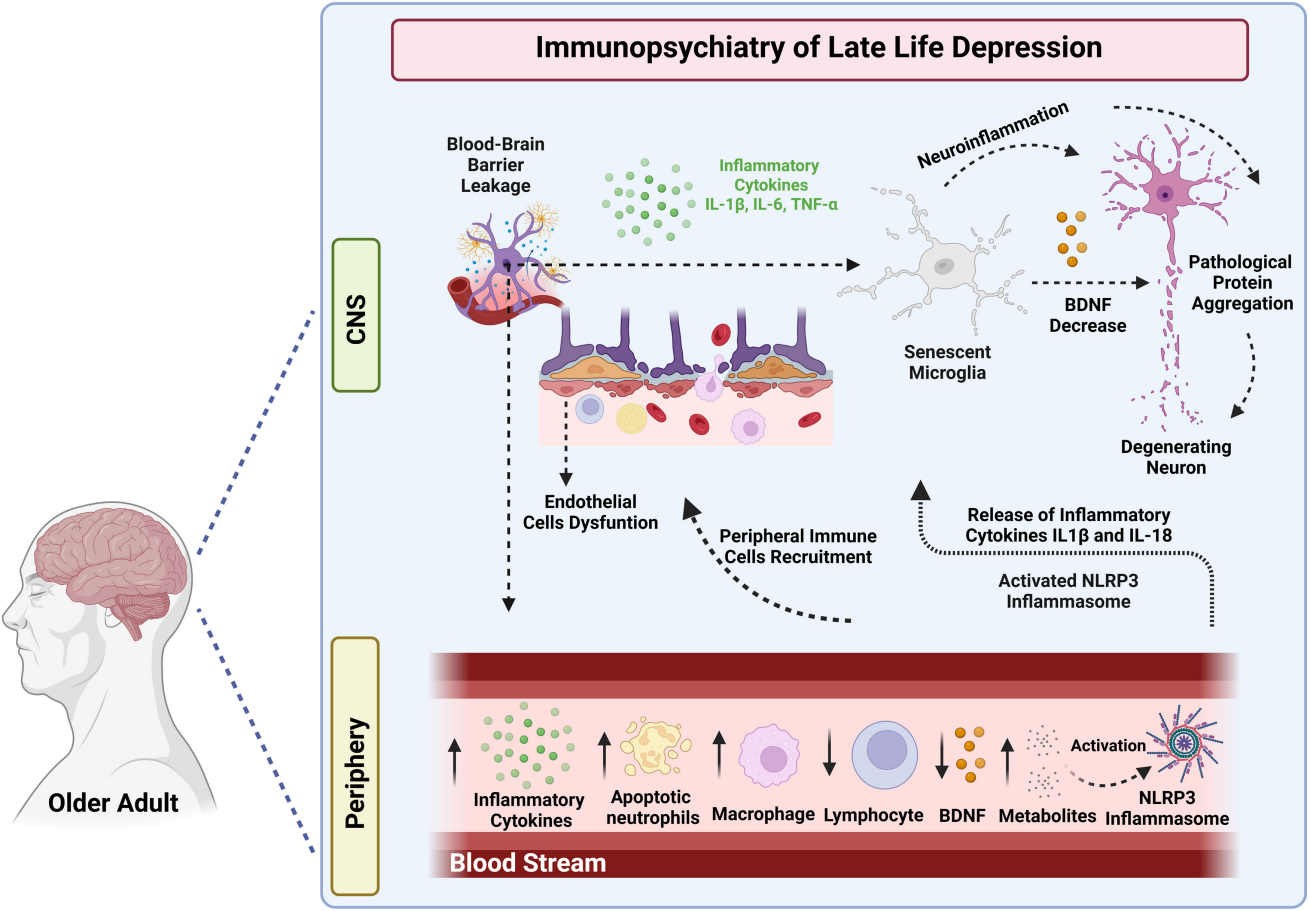
Potential immune/Inflammatory processes underlying late life depression pathogenesis. Ageing-related chronic low-grade inflammation (inflammaging) contributes to blood brain barrier (BBB) increased permeability and peripheral immune cells infiltration in the central nervous system resulting in senescent microglial activation, and neuroinflammation. Microglial activation and systemic inflammation also result in reduction of brain neurotrophic factor (BDNF) levels, all of which ultimately leading to pathological protein aggregation and neuronal death. These inflammatory events may underlie late life depression pathophysiology. Furthermore, older adults frequently present metabolic disorders, which contribute to the accumulation of endogenous metabolites including cholesterol crystals, urate crystals and lipotoxic ceramides that can activate the NOD-, LRR-, and Pyrin domain-containing protein 3 (NLRP3) inflammasome. The activation of NLRP3 inflammasome promote the release of inflammatory cytokines interleukin-IL (IL1-β) and IL-18, and, hence, a cascade of pro-inflammatory events that may also contribute to late life depression onset and progression. IL-6- Interleukin-6; TNF-α - tumour necrosis factor alpha. Created in BioRender.

Further discussion on the immune basis of LLD appears in section 3. Fig. 1 shows potential immune/inflammatory processes that may underlie LLD onset and progression.

### The vascular component of late-life depression

The vascular depression hypothesis proposes that cerebrovascular diseases, mainly small vessel ischaemic disease, may predispose, precipitate, and/or perpetuate depressive symptoms in late life. Aizenstein and coworkers (2016) proposed criteria for depression due to a vascular component, also called ‘vascular depression’: (i) depression occurring in an elderly person; (ii) absence of family history of depression; (iii) clinical symptoms marked by loss of energy, impaired motivation, anhedonia, subjective feelings of sadness, limited insight; (iv) cognitive dysfunction characterised by impaired executive function, processing speed and visuospatial skills; (v) presence of meaningful cerebrovascular risk factors; (vi) neuroimaging data confirming cerebrovascular disease; (vii) evidence of vascular pathology in elderly subjects with or without cognitive impairment (Aizenstein *et al*., 2016). While approximately one third of patients develop depression after a stroke (Ozkan *et al*., 2025), the concept of ‘vascular depression’ encompasses a broader group of patients with no history of transient ischaemic attack (TIA) or stroke or TIA but established cardiovascular risk factors for cerebrovascular disease and white matter changes indicative of small vessel disease. Importantly, ‘vascular depression’ is usually associated with LOD (Empana *et al*., 2021). However, white matter changes are one of the most frequent neuroimaging findings in adults with depression, indicating that microvascular changes are not restricted to LOD and can be associated with depression earlier in life (Wang *et al*., 2014).

Several pathogenic factors have been implicated in ‘vascular depression’, such as hemodynamic changes, neurovascular pathology, disorders of the glutamatergic system, mitochondrial dysfunction, lipid dysmetabolism, deficits of neurotrophic factors, and pro-inflammatory changes (Empana *et al*., 2021; Jellinger, 2021, 2022). Cerebrovascular risk factors, like diabetes and obesity, may promote a chronic pro-inflammatory profile. This enhanced peripheral inflammation may cause endothelial dysfunction and subsequent blood-brain barrier (BBB) disruption and microglia activation (Antonio L. Teixeira *et al*., 2025b). Activated microglia can produce a series of cytokines in the CNS, contributing to further changes in the endothelial cells and BBB, also affecting the function of neural circuits with the development of behavioural and cognitive symptoms (Mayer & Fischer, 2024).

### Late-life depression and neurodegeneration

There is a bidirectional relationship between depression and neurodegenerative diseases. While depression can be seen as a well-established risk factor for dementia, including Alzheimer’s disease (AD) and vascular dementia, it is also frequently comorbid with these conditions (Antonio Lucio Teixeira *et al*., 2025). Moreover, LLD and AD share clinical (e.g., cognitive symptoms) and neurobiological (e.g., decreased hippocampus volume) features (Antonio L. Teixeira *et al*., 2025a). Different models have been proposed to explain the bidirectional association between depression and AD, the prototype of neurodegenerative disease. More specifically, it has been postulated that EOD, especially with persistent or severe symptoms, should be regarded as a risk factor for AD, whereas LOD is a prodrome of AD (Huang *et al*., 2024).

From a mechanistic perspective, in addition to shared genetic liabilities, depression could contribute to the pathological aggregation of peptides, especially amyloid beta (Harrington *et al*., 2015). More likely, depression contributes to an inflammatory milieu in the CNS, influencing brain structure and function through the generation of toxic oxidative stress products, damage to neural and glial cells, and increased BBB permeability (Hayley *et al*., 2021). The combination of increased levels of inflammatory cytokines both locally and systemically with BBB permeability can lead to CNS immune activation and further lesion, perpetuating a damage cycle that can eventually progress to dementia. Antidepressant treatment of LLD can improve cognition, raising the possibility of prevention and/or attenuation of neurodegenerative changes (Ainsworth *et al*., 2024). Theoretically, this antidepressant effect could be mediated through anti-inflammatory mechanisms (Bajaj and Mahesh, 2024) . Neuroprotective effects were reported in APP/ PS1 transgenic mice, a model of AD, treated with antidepressants such as fluoxetine, a SSRIs (Huang *et al*., 2018; Zhou *et al*., 2019). Fluoxetine-related neuroprotection in AD seems to be, at least in part, mediated by the inhibition of the NF-κB/TLR4/NLRP3 signalling pathways and resultant release of pro-inflammatory cytokines like IL-1β and TNF-α (Bougea *et al*., 2024).

## Immunopsychiatry of LLD: the convergence of depression- and age-related inflammatory changes

**Figure 2.**
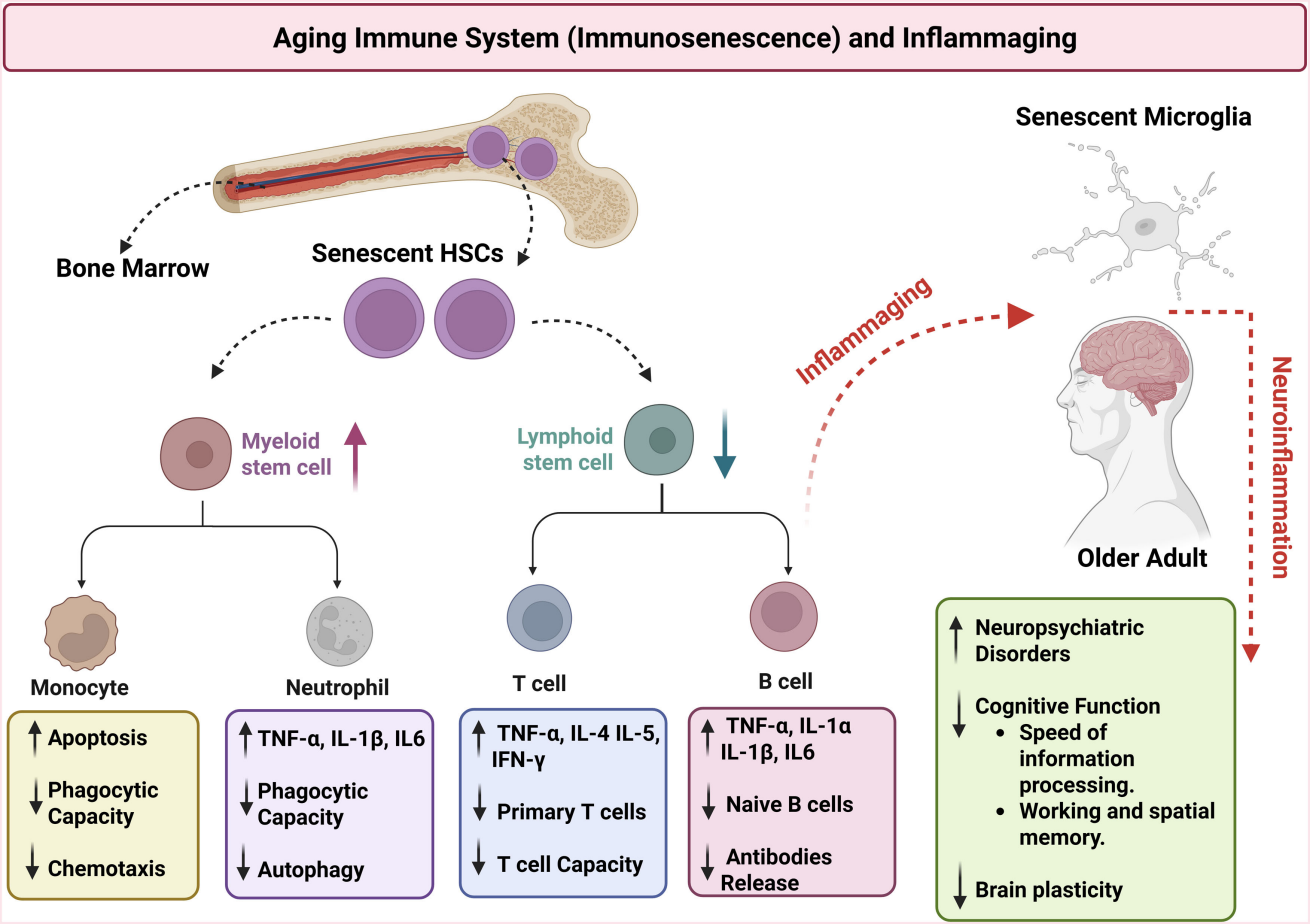
Age-related immune changes (immunosenescence) and systemic chronic low-grade inflammatory state or ‘inflammaging’. The ageing immune system is characterised, among others, by senescence of haematopoietic stem cells (HSCs) leading to an increased number of myeloid cells and decreased T and B lymphocytes, blunted antibody memory responses, impaired phagocytic and autophagy functions and antigen presentation, as well as increased levels of inflammatory mediators like interleukin-IL (IL1-β) and tumour necrosis factor alpha (TNF-α). ‘Inflammaging’ in the context of immunosenescence have been implicated in the development and progression of neuropsychiatric disorders and cognitive dysfunctions. IL-4- Interleukin-4; IL-5- Interleukin-5; IL-6- Interleukin-6; IFN-γ- interferon gamma. Created in BioRender.

The ageing immune system (immunosenescence) refers to remodelling humoral and cellular changes implicated in the development of most (if not all) age-related diseases. Ageing affects both innate and adaptive immune responses, ultimately contributing to a systemic chronic low-grade inflammatory state or ‘inflammaging’ (Cisneros *et al*., 2022). Among others, age-related immune changes include thymic involution, decreased number of T and B lymphocytes, blunted antibody memory responses, impaired phagocytic functions and antigen presentation, as well as increased levels of inflammatory mediators (Thomas *et al*., 2020; Teissier *et al*., 2022). ‘Inflammaging’ in the context of immunosenescence have been implicated in several pathological conditions, including neurodegenerative diseases and mood disorders (Fig. 2) (Barbé-Tuana *et al*., 2020; Antonio L. Teixeira *et al*., 2025b).

Although hypothalamic-pituitary-adrenal axis dysfunction, vascular risk factors, and deficits in neurotransmitter signalling have been implicated in the pathogenesis of depression, including LLD, its neurobiological basis remains unclear. Over the past two decades, immune/inflammatory processes have also been associated with depression pathogenesis in pre-clinical and clinical studies (Köhler *et al*., 2018; Yin *et al*., 2023). For instance, systemic or central administration of lipopolysaccharide (LPS), a potent inductor of immune response, have been widely employed as a model of depressive-like behaviours in rodents (Yin *et al*., 2023). In clinical settings, a meta-analysis of 82 case-control studies reported increased circulating levels of the inflammatory cytokines TNF-α and IL-6 in patients diagnosed with MDD compared to healthy controls (Köhler *et al*., 2018). Cytokines play a pivotal role in the regulation of inflammatory response and have been implicated in intercellular communication, such as glia-neuron interaction, and neuronal activity (Barbosa *et al*., 2014; Suchting *et al*., 2023). As for depression in adults, cytokines have been associated with LLD onset and progression.

Cross-sectional studies have reported increased levels of inflammatory cytokines TNF-α, IL-1β, and IL-6 in the serum of patients with LLD (Penninx *et al*., 2003; Tiemeier *et al*., 2003; Thomas *et al*., 2005; Bremmer *et al*., 2008). Similar findings were observed in longitudinal studies (Stewart *et al*., 2009; Kim *et al*., 2018). A two-year follow-up study with 521 Korean individuals aged ≥65 years showed a significant association between higher serum levels of IL-1β and IL-8 and LLD at baseline, independent of confounding variables like sex, cognitive performance, disability, lifestyle, and vascular risk factors. Both LLD at baseline and incident on follow-up were significantly associated with increased levels of IL-1β, IL-6, and IL8 at follow-up (Kim *et al*., 2018). Interestingly, increased inflammatory cytokine levels at baseline did not predict incident LLD, suggesting that cytokine changes could be secondary to the development of depression but not their main driver (Kim *et al*., 2018). A six-year longitudinal community study also showed that baseline IL-6 levels did not predict depressive symptoms on follow-up in a cohort of 263 healthy older adults (Stewart *et al*., 2009). Conversely, the Sydney Memory and Aging study reported that two-year incident depressive symptoms were associated with increased IL-18 levels at baseline in non-depressed and non-demented elderly participants aged 70 – 90 years (Baune *et al*., 2012). Furthermore, higher levels of IL-1 receptor antagonist (IL-1ra) at baseline were capable of predicting the development of LLD over a six-year follow-up (Milaneschi *et al*., 2009). Differences in the questionnaires and scales for evaluating depression, immunoassays for assessing biomarkers, age range of patients, and time-points of interviews may have contributed to the discrepancies found in these studies. Therefore, it remains to be defined whether elevated levels of inflammatory cytokines precede LLD onset (Kim *et al*., 2018), cytokines increase as a response to LLD, or both can coexist, reflecting the intrinsic heterogeneity of LLD. Further prospective studies are warranted to clarify this issue.

IL-6, known to be increased in ageing (Martínez-Cengotitabengoa *et al*., 2016), MDD and LDD (Kim *et al*., 2018), is the primary cytokine responsible for inducing the hepatic synthesis and release of acute phase proteins (APPs) such as CRP, pre-albumin, and albumin (Bode *et al*., 2012). Among these APPs, CRP has been the most extensively investigated as an inflammatory marker in LLD. The Atherosclerosis Risk in Communities (ARIC) Study conducted a 21-year longitudinal study with 4,614 participants and demonstrated that individuals who maintained elevated plasma levels of CRP over the study period had greater depression symptoms as older adults (Sonsin-Diaz *et al*., 2020). This observation supports the concept that sustained chronic (or repeated) inflammation for years or decades may contribute to the development of depression later in life (Sonsin-Diaz *et al*., 2020). In this line, the Netherlands Study of Depression in Older Persons (NESDO) showed that, after adjustment for demographics, health indicators, and medication use, higher plasma levels of CRP were associated with LOD but not EOD, suggesting a distinct etiopathogenetic profile depending on depression onset (Rozing *et al*., 2019). Prospective, large cohort studies with older participants also revealed that inflammatory dysregulation, including elevated CRP and IL-6 levels, alongside age-related metabolic changes (e.g., high triglycerides and glucose levels and lower levels of HDL cholesterol) were associated with more severe depression and worse clinical prognosis (de la Torre-Luque *et al*., 2019; Kokkeler *et al*., 2022). Finally, a study assessing 22 senescence-associated secretory phenotype (SASP)-related proteins in the plasma of 111 older adults found that patients with LLD had significantly higher SASP index compared to non-depressed controls, after controlling for age, sex, medical comorbidities, and cognitive performance (Diniz *et al*., 2017). Altogether, these results indicate the unequivocal association between LLD and a low-grade systemic inflammation.

In addition to systemic alterations, peripheral immunosenescence may cause microglial activation and neuroinflammation, which in turn may contribute to LLD (Ishizuka *et al*., 2024). Senescent/dystrophic microglia and increased inflammatory mediators such as TNF-α, IL-1β, and IL-6 have been detected in the aged brain (Streit *et al*., 2004; Wong, 2013; Ishizuka *et al*., 2024). Studies using positron emission tomography (PET) imaging have shown increased [^11^C]PK11195 binding in the anterior cingulate cortex and hippocampus of individuals with MDD (Holmes *et al*., 2018) and LLD (Su *et al*., 2016). As [^11^C]PK11195 is a radioligand that binds to the translocator protein (TSPO), a receptor expressed on activated microglia, these neuroimaging findings corroborate neuropathological data connecting neuroinflammation and depression. Particularly in LLD, microglial reactivity was associated with increased peripheral levels of CRP, supporting the link between ageing-related systemic and brain inflammation in LLD (Su *et al*., 2016). Furthermore, microglial activation and neuroinflammation have been implicated in decreasing BDNF signalling during ageing (Wu *et al*., 2020). Lower serum and CSF levels of BDNF have been associated with LLD and worse clinical outcomes (Diniz *et al*., 2014; Woods *et al*., 2021). Upon binding to high-affinity tropomyosin-associated kinase family (Trk) receptors, BDNF play critical roles in synaptic plasticity and neuronal growth, survival and differentiation (Xia *et al*., 2023). Pharmacological and non-pharmacological approaches, including physical exercise and diet, that have the potential to enhance systemic and brain BDNF levels in older individuals are promising therapeutic strategies for LLD (Azevedo *et al*., 2022; Pereira *et al*., 2013).

Another immune/inflammatory pathway that may underlie LLD onset and progression is the NOD-, LRR-, and Pyrin domain-containing protein 3 (NLRP3) inflammasome, composed of NLRP3, apoptosis-associated speck-like protein containing CARD (ASC), and caspase-1 (de Miranda *et al*., 2024). NLRP3 inflammasome drives innate immune responses through the release of the inflammatory cytokines IL-1β and IL-18, with the former being consistently implicated in MDD and LLD (Xia *et al*., 2023; Ishizuka *et al*., 2024). Metabolic diseases, including obesity and insulin resistance/diabetes, are common during ageing and have a reciprocal interaction with LLD (Martins *et al*., 2022; Dias *et al*., 2024). The pathological accumulation of endogenous metabolites, such as reactive oxygen species, cholesterol crystals, urate crystals and lipotoxic ceramides, can activate the NLRP3 inflammasome, leading to the release of cytokines and, hence, a cascade of pro-inflammatory events (Stienstra *et al*., 2011; Grant and Dixit, 2013; Lee *et al*., 2013; Youm *et al*., 2013; Xia *et al*., 2023). The combination of metabolic diseases and inflammation has been associated with severe depressive symptoms in older individuals (de la Torre-Luque *et al*., 2019; Kokkeler *et al*., 2022). Conversely, antidepressant treatments inhibited NLRP3 inflammasome in MDD, as observed by decreased serum levels of IL-1β and IL-18 and decrease of NLRP3 and IL-1β (p17) protein expression. Monitoring NLRP3 inflammasome components may have clinical value in drug selection and regulating the NLRP3/ASC/caspase-1/IL-1β/IL-18 pathway might be a promise therapeutic target for the development of novel antidepressant drugs (Alcocer-Gómez *et al*., 2017; Xia *et al*., 2023; de Miranda *et al*., 2024). Large population clinical studies addressing the potential link between NLRP3 inflammasome and LLD may lead to a deeper understanding of LLD neurobiological basis, better stratification of patients, and improvement of treatment outcomes.

As the body ages, haematopoietic stem cells (HSCs) tend to differentiate into myeloid cells, limiting the maturation of lymphoid progenitors of T and B cells. During ageing, there is an increased recruitment of inflammatory monocytes into the brain (Martin *et al*., 2017). Interestingly, the age-related cognitive decline was associated with increased MCP-1/CCL2 levels, a chemokine involved with monocyte recruitment (Bettcher *et al*., 2019), and higher levels of this chemokine were observed in LLD (Bocharova *et al*., 2025). In addition, brains of depressed suicides show significantly more infiltrated monocytes than healthy controls (Torres-Platas *et al*., 2014). The inflammatory profile of recruited monocytes into the brain may contribute to mood and cognitive changes observed in depression through activation of microglia, neurochemical changes (reduced monoamines and increased glutamate), and reduced brain neuroplasticity.

The aged-related inflammatory environment also contributes to immunosenescence of neutrophils, leading to cell metabolism dysregulation and increased neutrophils apoptosis (Dubey *et al*., 2016). In MDD patients, neutrophil to lymphocyte ratio, platelet to lymphocyte ratio, and monocyte to lymphocyte ratio are elevated (Demir *et al*., 2015; Demircan *et al*., 2016; Bulut *et al*., 2021) and may hold clinical value as low-cost and easily accessible inflammatory markers of MDD development and progression (Kayhan *et al*., 2017; Wei *et al*., 2019). However, their clinical potential in LLD remains to be further explored.

In addition to myeloid cells, aged circulating lymphocytes may also contribute to physiopathology of LLD. Human ageing and mood disorders lead to the expansion of senescent T cells (ex., CD28^null^ T cells), associated with poor immune responses and cognitive impairment (Bauer and Teixeira, 2019). These aged T cells are defined as senescent and display various cytotoxic and inflammatory properties, which may contribute to inflammaging by secreting large amounts of pro-inflammatory cytokines interferon (IFN)-γ, TNF-α, IL-1β, and IL-6 upon stimulation. The underlying mechanism by which senescent T cells modulate cognition is largely unknown, but there is preliminary evidence suggesting that senescent effector memory T cells can activate microglia (Ritzel *et al*., 2016). Patients with depression or LLD had leukocytes with shortened telomeres than age-matched controls, especially those with a more severe depressive episode (Mendes-Silva *et al*., 2021). These changes support the notion that neuropsychiatric disorders are characterised by premature ageing. Although there is a lack of information regarding the role of aged T cells in LLD, one study reported the accumulation of peripheral senescent T cells (CD57^+^CD28^null^) in older hip fracture patients who developed depressive symptoms (Duggal *et al*., 2014).

Taken together these studies provided compelling evidence of the involvement of immunosenescence and inflammaging in the pathophysiology of LLD (Table 1), supporting the concept that targeting ageing-associated immune/inflammatory changes might be a promising therapeutic approach for LLD.

**Table 1.** Clinical evidence of inflammatory markers in late life depression

Reference	Study Design	Experimental Population	Inflammatory Markers	Markers Measurement	Main Findings
Dentino *et al*. (1999)	Cross- sectional	1686 community-dwelling people, from either sex, aged 70 years and older	IL-6	Plasma ELISA	After controlling for age, race, and sex, higher levels of IL-6 was correlated with LLD
Penninx *et al*. (2003)	Cross- sectional	3024 well-functioning older adults, 70-79 years of age, participating in the Health, Aging and Body Composition study	IL-6, TNF-α, CRP	Plasma ELISA	145 depressed subjects had higher plasma levels of IL-6, TNF-α and CRP compared with 2879 nondepressed subjects
Tiemeier *et al*. (2003)	Cross- sectional	3884 adults at age 60 and older were evaluated. Plasma levels of inflammatory markers were compared between 263 cases with depressive symptoms (106 with depressive disorders) and 461 randomly selected reference subjects	CRP, IL-6	PlasmaCRP- Nephelometric method; IL-6- ELISA	Higher levels of IL-6 were associated with depressive disorders
Thomas *et al*. (2005)	Cross- sectional	3 groups of subjects older than 60 years: 19 subjects with MDD, 20 subjects with subsyndromal depression, and 21 healthy controls	IL-1β	Serum ELISA	LLD patients presented higher serum levels of IL-1β, which were correlated with depression severity
Bremmer *et al*. (2008)	Cross- sectional	1285 participants of the Longitudinal Aging Study Amsterdam, from either sex, aged 65 and over	IL-6, CRP	Plasma, ELISA	High plasma level of IL-6, but not CRP, was associated with LLD, independent of age, chronic diseases, cognitive functioning and anti-depressants
Stewart *et al*. (2009)	Longitudinal- Six-year follow-up	263 healthy, older men and women enrolled in the Pittsburgh Healthy Heart Project	IL-6, CRP	SerumCRP- Nephelometric method; IL-6- ELISA	Path analyses revealed that baseline depressive symptoms was a predictor of 6-year change in IL-6 serum levels
Milaneschi *et al*. (2009)	Longitudinal- Six-year follow-up	991 participants, ages 65 years and older enrolled in the InCHIANTI study	CRP, IL-1β, IL-6, TNF-α, IL-1ra, IL-6, IL-6R, IL-18	Serum ELISA	In older adults, high serum levels of IL1-ra were associated with higher risk of developing depressive symptoms over time
Diniz *et al*. (2010)	Cross-sectional	28 anti-depressant free patients with LLD, from either sex, compared with 39 age and sex-matched healthy elderly subjects	TNF-α, sTNFR1 and sTNFR2	Plasma ELISA	Patients with LLD showed increased plasma level of sTNFR2 compared with controls
Baune *et al*. (2012)	Longitudinal- Two-year follow-up	1037 non-demented community-dwelling elderly participants aged 70-90 years enrolled in the Sydney Memory and Aging Study	CRP, IL-1β, IL-6, IL-8, IL-10, IL12p70, PAI-1, TNF-α, sVCAM-1	Serum ELISA CBA	IL-6 and IL-8 were associated with current depressive symptoms; IL-8 were also associated with first onset of depressive symptoms; PAI-1 could be a marker of remitted depression
Teunissen *et al*. (2016)	Cross-sectional	350 older adults diagnosed with MDD in the last six months and 128 non-depressed controls from the Netherlands Study of Depression in Older persons	GDF-15	PlasmaNovel automated assay on Abbott Architect	GDF-15 was not an independent inflammatory marker for LLD
Su *et al*. (2016)	Cross-sectional	5 older adults diagnosed with MDD and 13 sex and age-matched haealthy controls enrolled in the Neuroimaging of Inflammation in Memory and Other Disorders (NIMROD) study	[^11^C]PK11195, a radioligand that selectively binds to TSPO, a receptor expressed on activated microglia	In vivo brain PET imaging	LLD was associated with increased [^11^C]PK11195 binding compared with controls in brain regions associated with depression, such as subgenual anterior cingulate cortex, and hippocampus
Diniz *et al*. (2014)	Cross-sectional	25 antidepressant-free patients with LLD (10 LLD + MCI and 15 LLD + NCD) and 25 age and sex-matched healthy older adults	BDNF	CSF ELISA	LLD + MCI patients showed lower CSF BDNF levels compared to LLD + NCD and healthy controls; Lower CSF BDNF levels correlated with more severe depressive symptoms and with worse cognitive performance
Diniz *et al*. (2016)	Cross-sectional	44 older adults age 65 years or older with remitted LLD and 31 age and sex-matched healthy older adults	Proteomic analysis of 232 proteins in the plasma	Plasma Multiplex immunoassay	Plasma levels of C-peptide, FABP-liver, and ApoA-IV were capable of accurately discriminate LLD from control subjects
Diniz *et al*. (2017)	Cross-sectional	80 older adults with LLD and 31 age and sex-matched healthy older adults	A panel of 22 senescent-associated secretory phenotype (SASP)-related proteins	Plasma Multiplex immunoassay	LLD patients displayed an increased SASP index, which were associated with higher medical comorbidity and worse cognitive function
Charlton *et al*. (2018)	Cross-sectional	24 individuals with LLD and 34 healthy older adults	IL-1β, IL-6, TNF-α	Plasma/serum ELISA	High levels of IL-6 were associated with cognitive decline in LLD
Kim *et al*. (2018)	Longitudinal- Two-year follow-up	732 Korean people aged 65+ were evaluated at baseline and 521 were followed over a 2 year period and incident depression was ascertained	IL-1 α, IL-1β, IL-6, IL-8 and TNF-α	Serum ELISA	Prevalent depression at baseline was significantly associated with higher levels of IL-1β and IL-8 in the follow-up; Incident depression was associated with increases in IL-1β, IL-6, and IL-8 levels during the follow-up
Rozing *et al*. (2019)	Cross-sectional/ Longitudinal- Two-year follow-up	350 patients, all aged 60 and older, with a depressive episode in the previous 6 months: 119 with LLD and 231 with EOD enrolled in the Netherlands Study of Depression in Older Persons (NESDO)	CRP, IL-6, NGAL, GDF15,	PlasmaELISACRP- high-sensitivity immunoturbidimetric assay	High plasma CRP levels were more strongly associated with LLD than EOD, suggesting a distinct inflammatory profile for LLD
de la Torre-Luque *et al*. (2019)	Longitudinal- Ten-year follow-up	1536 older participants (56.58% women; mean age at baseline = 61.73 years)	CRP, Fibrinogen	Plasma --------	High-symptom trajectory showed the greatest inflammation profile score and high metabolic risk
Sonsin-Diaz *et al*. (2020)	Longitudinal- Twenty one-year follow-up	4,614 participants were included (mean age: 75.5 years; 59% female; mean follow-up time: 20.7 years]). Participants with suspected depression during midlife were excluded	CRP	Serum Immunoturbidimetric assay	Elevated CRPLevels during the whole study period was associated with increased risk for LLD
Nie *et al*. (2021)	Cross-sectional	23 LLD, 23 aMCI and 23 healthy older controls	Panel of 29 cytokines	Plasma Luminex assays	LDD patients exhibited increased levels of IL-6 and reduced levels of CXCL11 and CCL13compared with aMCI as well as reduced levels of CXCL16 compared with healthy controls
Kokkeler *et al*. (2022)	Longitudinal- Two-year follow-up	364 patients all aged 60 and older, diagnosed with MDD enrolled in the Netherlands Study of Depression in Older persons (NESDO) study	CRP, IL-6, GDF-15, NGAL	PlasmaCRP- high-sensitivity immunoturbidimetric assay; IL-6 and NGAL – ELISA; GDF15 - automated assay on Abbott Architect	Patients with LDD presented metabolic and inflammatory dysregulation, which were associated with more severe depression and a worse prognosis
Lozupone *et al*. (2023)	Longitudinal- Eight-year follow-up	1479 participants, all aged 65 and older, enrolled in the Salus in Apulia Study	CRP, IL-6, TNF-α	SerumCRP- high-sensitivity immunoturbidimetric assay; IL-6 and TNF-α- ELISA	High leves of IL-6, among others, were associated with LLD increased risk of all-cause mortality
Ho *et al*. (2024)	Cross-sectional	52 non-demented MDD patients, comprising 25 with MCI, were recruited along with 10 control healthy subjects.	TNF-α	BloodChemiluminescent immunoassay using IMMULITE 1000 (SIEMENS).	MCI MDD patients showed increased TNF-α levels and reduced hippocampal volume, indicating a potential association between peripheral inflammation and structural brain alterations in LLD
Dias *et al*. (2024)	Cross-sectional	25 elderly female patients with MDD and 19 age-matched female healthy controls	IL-4, IL-6, IL-10, IFN-γ and TNF-α	Plasma Luminex	Elderly female patients exhibited higher plasma IL-6 and IL-4 levels compared to controls
Bocharova *et al*. (2025)	Longitudinal- Three-year follow-up	136 patients with LLD (PRODE cohort) compared with 103 cognitively healthy non-depressed controls (COGNORM cohort)	IL-1β, IL-1ra, IL-6, IL-10, IL-17a, IL-18, IL-33, TNF-α, CD40L, IFN-γ, CCL-2 and CCL-4	Serum Multiplex immunoassay	Serum levels of IL-1ra, CCL-2, CCL-4, IFN-γ and IL-17a were higher in LLD patients compared to controls

aMCI- amnestic mild cognitive impairment, BDNF- Brain-Derived Neurotrophic Factor; CBA- cytometric bead array; CRP- C-reactive protein; CCL-2-CC chemokine ligand 2; CCL-4-CC chemokine ligand 4; ELISA - enzyme-linked immunosorbant assay; EOD- early-onset depression; FABP-liver - fatty acid binding protein – liver; GDF-15-Growth Differentiation Factor-15; IL-1β - interleukin (IL)-1beta, IL-1ra -IL-1 receptor antagonist (ra); IL-4- interleukin (IL)-4; IL-6- interleukin (IL)-6; IL-10- interleukin (IL)-10; IL-17a- interleukin (IL)-17a; IL-18- interleukin (IL)-18; Interferon-gamma (IFN-γ); LLD- late life depression; MDD- major depressive disorder; MCI- mild cognitive impairment; NCD - non cognitive decline; NGAL-neutrophil gelatinase-associated lipocalin; PAI-1 -plasminogen activator inhibitor-1; PET -positron emission tomography imaging; sVCAM-1- serum vascular adhesion molecule-1; TNF-α- tumour necrosis factor-alpha;, TSPO- translocator protein.

## Therapeutic opportunities in LLD: focus on inflammation

The DSM-5 criteria for depression are the same for younger and older adults. However, a patient with LLD tends to have more physical complaints such as fatigue, pain, multiple unexplained medical symptoms rather than a depressed mood and related subjective (e.g., sadness) symptoms (Hegeman *et al*., 2012). Despite these clinical and potential pathophysiological differences, the treatment of depression in older patients is also similar to the one applied to younger patients. The main treatments are depression-specific psychotherapies, like cognitive-behavioural and interpersonal therapies, and antidepressants. However, a significant proportion of patients with LLD have treatment-resistant depression (Steffens, 2024; 87). The mechanisms underlying this phenomenon are not clear (de Miranda *et al*., 2025), but seem to involve clinical (e.g., presence of multiple physical comorbidities) and neurobiological mechanisms. As discussed above, LLD has significant associations with cerebrovascular burden, neurodegenerative changes and exacerbated inflammatory profile. In this context, inflammation-related biomarkers and targets could help the identification, categorisation, and management of LLD.

But what are the sources or causes of this inflammatory profile in LLD? Increasing evidence suggests that ageing and illness-related changes in neuroendocrine function with dysregulation of the hypothalamic-pituitary-adrenal axis, diet/microbiota (e.g., western diet pattern), unhealthy behaviours (e.g., physical inactivity, smoking, social isolation), and ‘wear and tear’ of physiological systems driven by comorbidities are important drivers of inflammation (Bauer & Teixeira, 2019; Teixeira *et al*., 2022). Therefore, addressing diet, physical inactivity, use of drugs and optimal management of physical comorbidities can be relevant strategies to prevent and/or minimise the severity of LLD (Martins *et al*., 2021, 2022; Pearce *et al*., 2022; Barbosa *et al*., 2025). For instance, the Mediterranean diet, usually characterised by a higher intake of fruits, vegetables, whole grains, and good quality sources of protein (e.g., fish) and diets with lower ‘Diet Inflammatory Index’ have been associated with lower incidence of depression (Lassale *et al*., 2019). Moreover, microbiota-targeted interventions, such as symbiotics, prebiotics, and probiotics, seem to attenuate depression, at least in part, through inflammation-related mechanisms (Barbosa *et al*., 2025). One caveat is related to the fact that most of the available evidence in the field of nutrition psychiatry has been obtained in adult populations, and studies involving older adults and LLD are still scarce.

In addition to their effects on neurotransmitter systems, influencing the levels of monoamines and the expression of related receptors, antidepressants also have immunomodulatory actions (Tomaz *et al*., 2020). A recent meta-analysis including 839 patients confirmed that selective serotonin reuptake inhibitors (SSRIs) significantly reduce the circulating levels of IL-6 and TNF-α in patients with depression (Patel *et al*., 2024). While this decrease may be an indirect effect of depression improvement, SSRIs can directly influence immune cells number (decreasing their proliferative activity and increasing their apoptosis) and function (Szałach *et al*., 2019). It is therefore tempting to speculate that the therapeutic effects of antidepressants are partly mediated by their immunomodulatory/anti-inflammatory actions. This hypothesis is supported by studies showing that anti-inflammatory and immunomodulatory strategies, including non-steroidal anti-inflammatory and anti-cytokine antibodies, can improve depressive symptoms (Colpo *et al*., 2018; Köhler *et al*., 2018). However, in contrast with studies carried out in adult populations, clinical trials with celecoxib (Fields *et al*., 2012), naproxen (Fields *et al*., 2012), and aspirin (Berk *et al*., 2021) in LLD failed to demonstrate benefit of anti-inflammatory strategies. These data strengthen the view that LLD must be seen as a condition with unique pathophysiological mechanisms and, thus, a particular therapeutic response profile, not necessarily as an extension of adult depression. It is possible that inflammation-related biomarkers can help to define subtypes of LLD. Although previous attempts to differentiate clinical subtypes of depression in adults (e.g., atypical versus melancholic) based on inflammatory biomarkers have failed (Veltman *et al*., 2018), this hypothesis must be thoroughly considered in LLD where other biological players (e.g. neurodegeneration) play pivotal roles.

It is worth mentioning that neuromodulation methods, such as electroconvulsive therapy (ECT), transcranial magnetic stimulation (TMS), and vagus nerve stimulation, have shown promising results in the elderly population with depression (Steffens, 2024). Interestingly, it has been proposed that the observed therapeutic effects are partly mediated through immune/inflammatory mechanisms (Kranaster *et al*., 2018; Belge *et al*., 2021; Goerigk *et al*., 2021). For instance, Zhao *et al*. (2019) reported that a course of TMS for five times a week for four weeks significantly decreased the severity of depression symptoms in parallel with increase in BDNF levels, and decrease in IL-1β and TNF-α levels in patients with treatment-resistant LLD (Zhao *et al*., 2019). The evidence is less clear for ECT as cytokine levels and CRP were similar between remitters and non-remitters patients with LLD submitted to ECT (Carlier *et al*., 2019, 2022).

## Conclusion

LLD has unique clinical and biological features compared to depression in other periods of life. The recognition of its intrinsic pathophysiological heterogeneity, comprising multiple psychosocial and biological pathways, is an important step toward its proper management. Inflammatory mechanisms seem to play pivotal roles in LLD development and progression, directly influencing neural circuits subserving depressive symptoms and indirectly influencing other pathways, such as vascular and neurodegenerative ones. While the pathogenesis of the low-grade inflammation in LLD is still unclear, it is potentiated by the senescence of immune cells (that tend to assume a proinflammatory profile with ageing) and the burden of physical comorbidities (e.g., cardiovascular and metabolic diseases). These inflammatory mechanisms can be targeted and, if downregulated, can help to mitigate and/or prevent LLD. In this context, immune-based strategies may play a therapeutic role for LLD. Large-sample, randomised, double-blind clinical studies focusing on anti-inflammatory treatment for geriatric depression is urgent needed to deepen our understanding of the efficacy and safety of these therapies for LLD.

## Supporting information

Teixeira et al. supplementary material Teixeira et al. supplementary material

## Supplementary material

For supplementary material/s referred to in this article, please visit https://doi.org/10.1017/neu.2025.10019
