# Supplementary material for: Immunopsychiatry of late life depression: role of ageing-related immune/inflammatory processes in the development and progression of depression
Source: Acta Neuropsychiatr. 2025 Jun 13;37:e67. doi: 10.1017/neu.2025.10019 (PMC13130277; doi:10.1017/neu.2025.10019)
Supplement: Teixeira et al. supplementary material [file S0924270825100197sup001.docx]

Article Highlights

- LLD has unique clinical and biological features compared to depression in other periods of life.

- Low-grade systemic inflammation or inflammaging may underlie LLD onset and progression.
- Targeting aging-associated immune/inflammatory changes might be a promising therapeutic approach for LLD.
